# Ureteroscopic Resection of Ureteral Tumor

**DOI:** 10.1590/S1677-5538.IBJU.2019.0255

**Published:** 2020-02-20

**Authors:** Eclair Lucas, Luis César Zaccaro da Silva, Gilberto Saber

**Affiliations:** 1 Santa Casa de Misericórdia de Ribeirão Preto Departamento de Urologia Ribeirão PretoSP Brasil Departamento de Urologia, Santa Casa de Misericórdia de Ribeirão Preto Ribeirão Preto, SP, Brasil

## INTRODUCTION

Accounting for only 5% of all renal and urothelial tumors, upper tract urothelial carcinoma (UTUC) is a rare genitourinary malignancy. Although management guidelines for UTUC recommend radical nephroureterectomy (RNU) with resection of a bladder cuff as the ‘gold standard’ treatment, the solitary kidney status after this procedure may lead to higher rates of dialysis, cardiovascular morbidity, and overall mortality. In an effort to mitigate these attendant risks, ureteroscopy (URS) and laser photoablation represent a valid treatment option for these patients with high comorbidities and/or low-risk disease and willing to undergo an intensive surveillance program.

Minimally-invasive endoscopic management of UTUC was first suggested for imperative cases as chronic kidney disease, solitary kidney, bilateral UTUC, and the good results obtained in terms of cancer control lead clinicians to offer this approach also to elective cases (patients with normal contralateral kidney).

The endoscopic treatment of upper tract UTUC coincided with the development and refinement of percutaneous renal surgery, ureteroscopy, and laparoscopy. These techniques can now be combined to provide histologic diagnosis of filling defects of the upper urinary tract, remove small to even large intraluminal lesions, or remove the distal ureter or the entire kidney and ureter with endoscopes alone.

## CASE REPORT

We present the case of an 85-year-old male with asymptomatic gross hematuria for 1 month. The patient, despite advanced age, presented good performance status, and as comorbidities, presented cardiomyopathy, previous stroke (one year) and using Xarelto^®^ (rivaroxaban), AAS, simvastatin and digoxin.

In the requested imaging tests, with better accuracy, the MRI revealed a lesion in the upper right ureter of a superficial and non-invasive character. Ureteroscopy then revealed a typical urothelial, pedunculated lesion of approximately 1cm, and was then resected by endoscopic resection with YAG-Holmium laser, and extraction of the specimen with Dormia's basket.

The surgery was performed without any complications, with a total time of 30 minutes and the patient was discharged on the first postoperative day, with a double-j stent, and asymptomatic.

Anatomopathological examination revealed superficial urothelial carcinoma (pTa) grade II/(high grade).

